# Brain Structural and Functional Dissociated Patterns in Degenerative Cervical Myelopathy: A Case-Controlled Retrospective Resting-State fMRI Study

**DOI:** 10.3389/fneur.2022.895348

**Published:** 2022-06-15

**Authors:** Yi Zhou, Jiaqi Shi

**Affiliations:** Department of Orthopedics, Xiangyang Central Hospital, Affiliated Hospital of Hubei University of Arts and Science, Xiangyang, China

**Keywords:** fMRI, VBM, functional connectivity density, degenerative cervical myelopathy, gray matter atrophies

## Abstract

**Background:**

Previous studies have shown the whole-brain global functional connectivity density (gFCD) and gray matter volume (GMV) alterations in patients with degenerative cervical myelopathy (DCM). However, no study aimed to investigate the associations between the spatial patterns of GMV and gFCD alterations in patients with DCM.

**Methods:**

Structural data and resting-state functional MRI data of 35 DCM patients and 35 matched healthy controls were collected to assess their gFCD and GMV and investigate gFCD and GMV alterations in patients with DCM and their spatial pattern associations.

**Results:**

In our current study, significant gFCD and GMV differences were observed in some regions of the visual system, sensorimotor cortices, and cerebellum between patients with DCM and healthy controls. In our findings, decreased gFCD was found in areas primarily located at the sensorimotor cortices, while increased gFCD was observed primarily within areas located at the visual system and cerebellum. Decreased GMV was seen in the left thalamus, bilateral supplementary motor area (SMA), and left inferior occipital cortices in patients with DCM, while increased GMV was observed in the cerebellum.

**Conclusion:**

Our findings suggest that structural and functional alterations independently contributed to the neuropathology of DCM. However, longitudinal studies are still needed to further illustrate the associations between structural deficits and functional alterations underlying the onset of brain abnormalities as DCM develops.

## Introduction

Degenerative cervical myelopathy (DCM), characterized by acquired cervical spine stenosis during the aging process (1), is becoming increasingly prevalent due to global aging and lifestyle changes. DCM has also been considered the most frequently reported non-traumatic spinal cord dysfunction in clinical practice (2), which requires timely diagnosis and spinal canal decompression to relieve myelopathy before irreparable damage to the spinal cord (3, 4). Although the surgical indications for DCM are quite clear (5), it is still difficult for spine surgeons to decide whether patients with long-term myelopathy (i.e., lasting for years or decades) or mild myelopathy would benefit from decompression surgery (6). Subsequently, simple and accurate markers are needed to monitor the myelopathy progression and inform surgical decisions (7). In the past decade, researchers have relied on conventional cervical spine MRI to monitor and determine spinal cord structural changes in DCM patients, e.g., high signal intensity on T2-weighted MR (8, 9). Its utility remains controversial as the information obtained from the spinal cord area is limited, i.e., the small cross-sectional area (10–12). Therefore, researchers set their sights on resting-state functional MRI for exploring brain alterations in patients with DCM (13–15). Several studies reported that DCM is associated with significant brain structural and functional changes (13–24). It has been shown that patients with DCM exhibit a significantly decreased gray matter volume (GMV) in various cortical and subcortical brain regions. Notably, the frontal, sensorimotor, and occipital cortices, thalami, and cerebellum are key regions with GMV decrease in DCM patients (13, 17, 18, 20–23). Further, results from fMRI studies indicated that functional alterations, which are characterized by reduced functional activity in subcortical regions such as the basal ganglia and some regions of the limbic system, and concurrent hyper-functional activity in cortical regions were observed in patients with DCM (15, 17–19, 25, 26). They have also been shown to exhibit functional alterations within frontal, temporal, and parietal lobes, sensorimotor cortex, cingulate gyrus, occipital gyrus, hippocampus, and thalami regions (26–32).

Whole-brain measurement of voxel-based morphometry (VBM) is an unbiased and fully automated method for evaluating GMV alterations (33, 34). These GMV changes can serve as structural biomarkers for clinical and research applications (33, 35). Functional connectivity density mapping (FCDM) is a voxel-wise data-driven method used to determine the number of functional connections between a given voxel and other voxels in the whole brain (36, 37). FCDM measures global functional connectivity density (gFCD) (38–41), an indicator of the number of resting-state functional connections of a given voxel with all other voxels in the entire brain, reflecting the one-to-many relationship (42). Traditional functional connectivity methods mainly involve measuring the connectivity strength between two voxels, regions, or networks, thus only reflecting a one-to-one relationship (42–44). In contrast, gFCD reflects the aberrant functional activity from several perspectives compared with traditional functional connectivity methods (42). A recent study reported that gFCD is a key biomarker for various diseases (45). Although previous studies reported the aberrations in GMV and gFCD as important characteristics of DCM (18, 46, 47), the associations between the spatial distribution patterns of GMV and gFCD alterations remain unexplored.

Therefore, we adopted VBM and FCDM approaches in the present study to explore the alterations in GMV and gFCD and their spatial distribution patterns in patients with DCM. Several previous studies reported that DCM is associated with significant anatomical and functional brain aberrations (47, 48). Anatomical aberrations are mainly located within sensorimotor regions, whereas functional aberrations are mainly observed in frontal, sensorimotor, and default-mode network regions (16–19). Notably, spatial and temporal distributions of anatomical and functional alterations are not correlated, and the aberrant patterns are inconsistent, indicating relatively “distinct patterns” (48). This study explores whether gFCD and GMV are altered in patients with DCM compared with matched healthy controls. An analysis was conducted to explore whether gFCD and GMV were simultaneously affected in particular brain regions and the relationships between the spatial distributions and aberrant patterns (increased or decreased).

## Materials and Methods

### Subjects

This retrospective study adheres to the STROBE guidelines (49). Ethical approval for this study was obtained from the local institutional review board. All participants provided written informed consent before participating in the current study. A total of 35 right-handed DCM patients were recruited consecutively from 2014 to 2020 according to the following inclusion criteria: (1) Patients with explicit cervical MR evidence of spinal cord compression (from C1 to C7); (2) patients clinically diagnosed with cervical myelopathy, with MRI findings consistent with the level of clinical signs. The common presenting symptoms include neck pain or stiffness, pain, weakness, or numbness (paraesthesias) in the upper limbs, loss of manual dexterity (clumsiness), stiffness, weakness, or numbness of the lower limbs, gait imbalance or unsteadiness, and falls. Objective physical signs of myelopathy include upper motor neuron signs in the upper and/or lower limbs (1); (3) patients with no history of surgery at the spinal canal; (4) patients capable of finishing the fMRI scan; (5) patients with no history of other diseases; (6) patients with no alcohol and substance abuse history. A total of 35 healthy subjects with matching age, gender, and education were recruited through advertisements according to the following inclusion criteria: (1) Individuals with no MR or clinical evidence of myelopathy; (2) individuals with no other spinal or brain neurological disorders or systemic diseases; (3) individuals capable of finishing the fMRI scan; (4) individuals with no history of other diseases; (5) Individuals with no alcohol and substance abuse history.

### MRI Data Acquisition

MRI was performed using a 3.0-Tesla MR system (Discovery MR750, General Electric, Milwaukee, WI, USA). Tight but comfortable foam padding was used to minimize head motion, and earplugs were used to reduce scanner noise during MRI examination. Sagittal 3D T1-weighted images were acquired using a brain volume sequence with the following parameters: repetition time (TR) = 7.8 ms; echo time (TE) = 2.8 ms; inversion time (TI) = 440 ms; flip angle (FA) = 12°; field of view (FOV) = 256 × 256 mm; matrix = 256 × 256; slice thickness = 1 mm, no gap; and 188 sagittal slices. Resting-state fMRI data were acquired using a gradient-echo single-short Echo planar imaging sequence with the following parameters: TR/TE = 2,000/30 ms; FOV = 220 × 220 mm; matrix = 64 × 64; FA = 90°; slice thickness = 3 mm; gap = 0 mm; 48 interleaved transverse slices; and 180 volumes. All subjects were requested to keep their eyes closed, relax, minimize movement, think of nothing in particular, and not fall asleep during the scanning period. The images were evaluated right after the data acquisition by a senior radiologist, and subjects with low-quality structural or functional images were asked to retake the scan. Each patient was assessed by a senior orthopedic surgeon using the Japanese Orthopedic Association (JOA) score, which measures the sensorimotor function in patients with DCM.

### fMRI Data Preprocessing

Resting-state fMRI data were preprocessed using the Statistical Parametric Mapping software (SPM8; http://www.fil.ion.ucl.ac.uk/spm). The first 10 volumes of each subject were discarded to obtain the equilibrated signal and allow the participants to adapt to the scanning noise. The remaining 170 volumes were corrected for the acquisition time delay between slices. Realignment was then performed to correct for motion between time points. The fMRI data of all subjects were within the defined motion thresholds, namely, translational or rotational motion parameters below 2 mm or <2°. The frame-wise displacement, which indexes volume-to-volume changes in head position, was also determined. Several nuisance covariates, including the six motion parameters, their first-time derivations, and average BOLD signals of the ventricular and white matter, were regressed from the data. A recent study reported that the signal spike caused by head motion significantly contaminates the final resting-state fMRI results even after adjusting for the six motion parameters (50). Therefore, spike volumes were adjusted when the frame-wise displacement (determined using the FD Jenkinson method) of the specific volume exceeded 0.5. The datasets were then band-pass filtered in a frequency range of 0.01 to 0.08 Hz (51). Individual structural images were linearly co-registered with the mean functional image in the normalization step and then linearly co-registered to the Montreal Neurological Institute (MNI) space. Each filtered functional volume was then spatially normalized to the MNI space using the co-registration parameters and resampled into a 3-mm cubic voxel.

### GMV Calculation

The GMV of each voxel was determined using SPM12. Structural MR images were classified into gray matter (GM), white matter, and cerebrospinal fluid using the standard unified segmentation model. GM concentration images were non-linearly warped using the diffeomorphic anatomical registration through exponentiated lie algebra technique after the initial affine registration of the GM concentration map into the MNI space. The results were then resampled to a voxel size of 3 × 3 × 3 mm. The GMV of each voxel was obtained by multiplying the GM concentration map with the non-linear determinants derived from the spatial normalization step. The GMV images were further smoothed using a Gaussian kernel with 6 × 6 × 6 mm full-width at half maximum. The smoothed GMV maps were used for statistical analyses after spatial preprocessing.

### Determination of FCD

The FCD of each voxel was determined using a Linux script developed in-house according to the method described by Tomasi and Volkow (45). Pearson's linear correlation analysis was conducted to evaluate the functional connectivity strength between voxels (52). Voxel pairs with a correlation coefficient of *R* > 0.6 were considered significantly connected and used for subsequent analysis (38–40, 42, 43). The FCD calculations were restricted to the cerebral gray matter mask regions. The gFCD at a given voxel, x_0_, was computed as the global number of functional connections, k(x_0_), between x_0_ and all other voxels. This calculation was performed for all x_0_ voxels in the brain. The FCD maps were spatially smoothed using a 6 × 6 × 6 mm Gaussian kernel to minimize differences in functional brain anatomy across subjects.

### Statistical Analysis

Group differences in GMV and gFCD were compared in a voxel-wise manner using a two-sample *t*-test with age and gender as confounding variables. Multiple comparisons were corrected using the false discovery rate method with a corrected threshold of *P* < 0.05. Mean gFCD and GMV values of each cluster, with significant group differences in gFCD, were extracted for each subject and used for the region of interest (ROI)-based group comparisons. Group differences in GMV for each ROI were evaluated using two-sample *t*-tests after controlling for age and gender to explore if the regions with altered gFCD exhibited structural impairment. Pearson correlation analysis was conducted to explore the relationship between gFCD and GMV in each group and evaluate the associations between gFCD and clinical variables, including JOA scores and illness duration.

## Results

### Subject Demographics and Clinical Characteristics

The demographic and clinical characteristics of the subjects are summarized in Table 1. The results showed no significant gender or age differences between the two groups (*t* = 0.46, *P* = 0.53). A total of 35 patients underwent fMRI examinations, and the mean duration of illness was 32.4 ± 15.8 months. The mean preoperative JOA score was 11.2 ± 3.9.

**Table 1:** Demographic data of the two groups.

	**DCM (*n* = 35)**	**HC (*n* = 35)**	***P*-value**
Age (years)	54.27 ± 6.25	53.92 ± 7.28	0.53
Gender (F/M)	17/18	17/18	1
Education (years)	12.3 ± 2.17	12.2 ± 3.41	0.54
JOA	11.2 ± 3.91		
Duration of illness (month)	32.4 ±15.8		

*DCM, degenerative cervical myelopathy; HC, healthy controls; JOA, Japanese Orthopedic Association*.

### Distributed Specificity of gFCD Hubs in Patients and Controls

**Figure 1:**
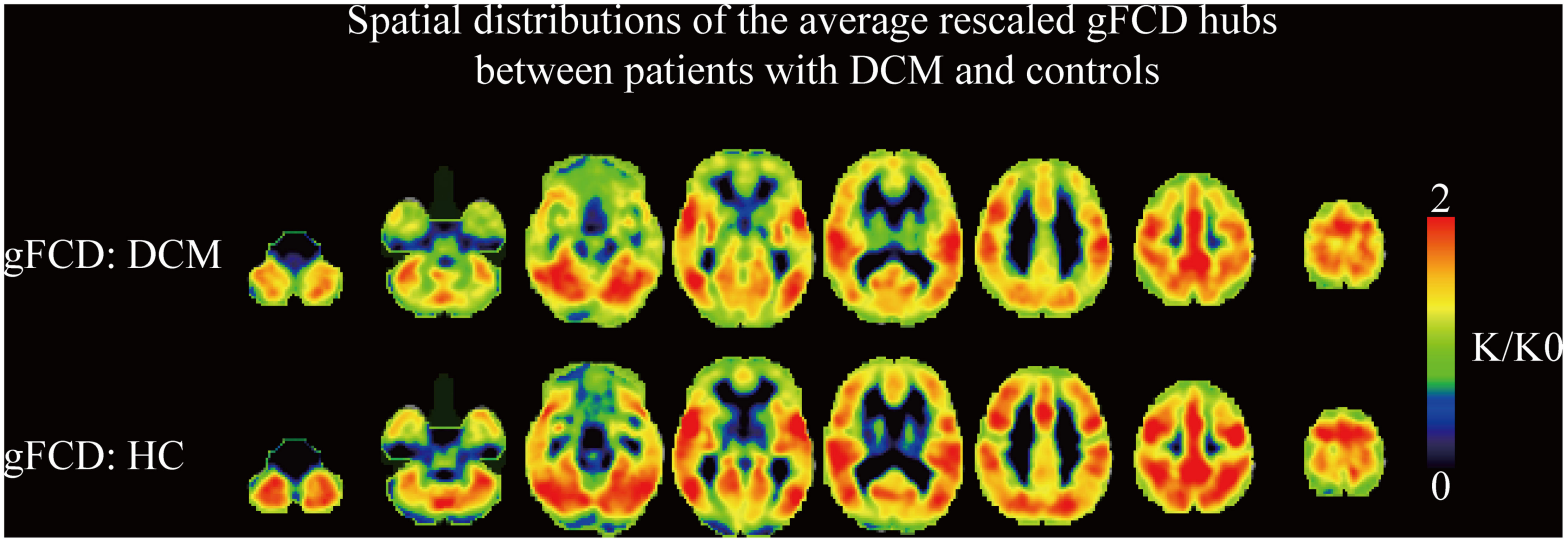
The spatial distributions of the averaged rescaled global functional connectivity density hubs between DCM patients and healthy controls. gFCD, global functional connectivity.

The results suggested that the strongest gFCD hubs in the two groups were mainly located in the precuneus, inferior parietal lobe, superior temporal gyrus, medial prefrontal cortex, and dorsal lateral prefrontal cortex. Most of the gFCD hubs were located in the default mode network and sensory cortices (Figure 1). The distribution of gFCD hubs was not significantly different between the two groups.

### gFCD Changes in Patients With DCM

**Figure 2:**
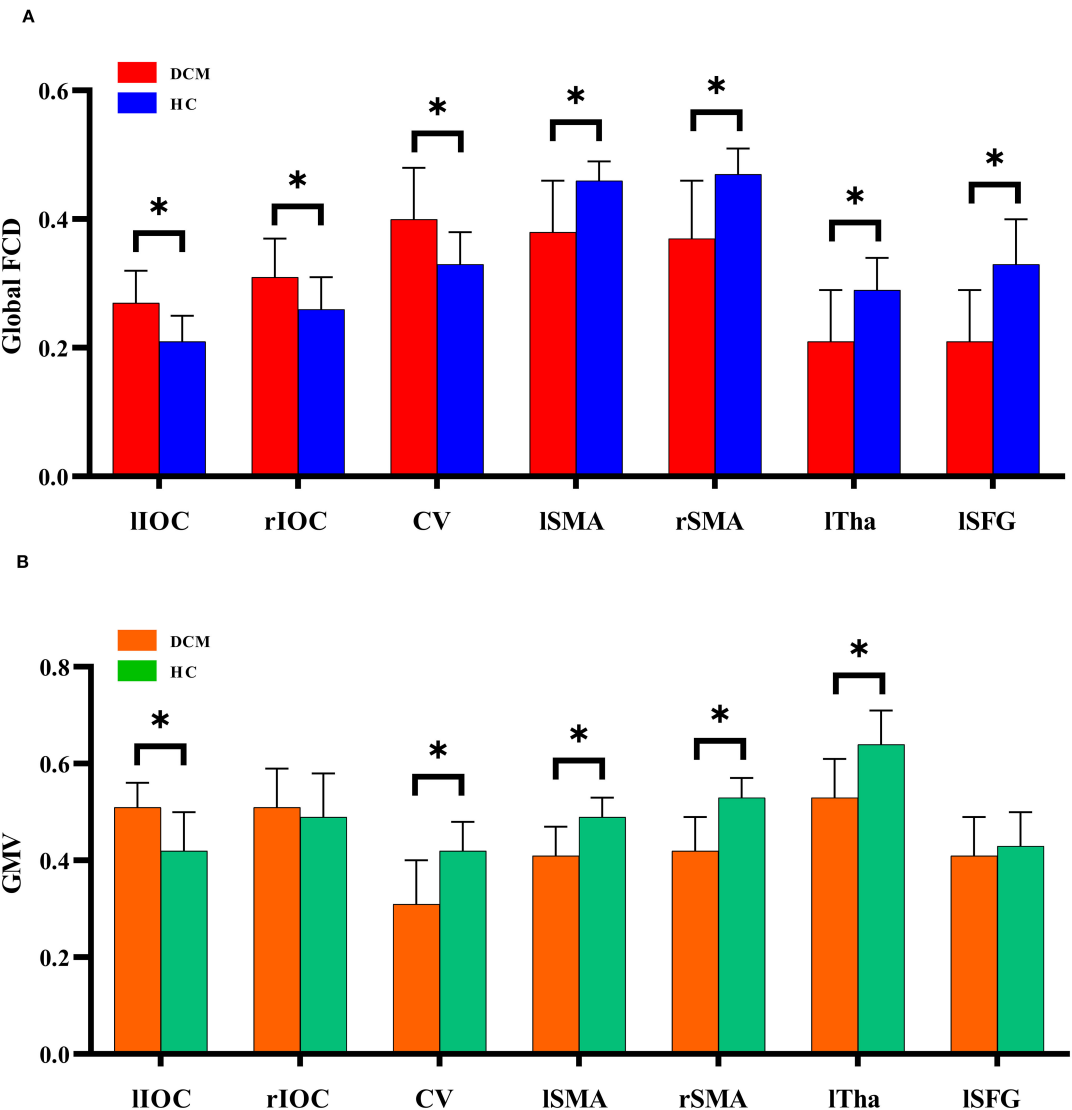
The global functional connectivity density differences between degenerative cervical myelopathy patients and healthy controls. IOC, inferior occipital cortex; CV, cerebellum vermis; SMA, supplementary motor area; Tha, thalamus; SFG, superior frontal gyrus; l, left; r, right. *P < 0.05.

Voxel-wise analysis showed significantly increased gFCD (i.e., *q*-value < 0.05, as corrected by the FDR method) in bilateral inferior occipital cortices and cerebellum (Figure 2A, Table 2). Significantly decreased gFCD (i.e., *q*-value < 0.05, as corrected by the FDR method) was observed in the sensorimotor cortex, including bilateral supplementary motor area (SMA), left thalamus, and left superior frontal gyrus in patients with DCM (Figure 2B, Table 2).

**Table 2:** Detailed information on intergroup global functional connectivity density (gFCD) differences.

**Brain regions**	**MNI coordinates**			**Peak intensity**	**Voxel size**
DCM > HC					
Left inferior occipital gyrus	−21	−81	−5	3.53	121
Right inferior occipital gyrus	24	−86	−6	3.42	72
Cerebellum vermis	1	−61	−24	3.38	88
DCM < HC					
Right SMA	5	7	56	−3.71	83
Left SMA	−3	6	52	−3.71	91
Left thalamus	−14	−17	16	−3.62	81
Left superior frontal gyrus	−32	14	53	−3.51	78

*DCM, Degenerative Cervical Myelopathy; HC, Healthy Controls; SMA, Supplementary Motor Area*.

### Gray Matter Volume Changes in Patients With DCM

Decreased GMV was observed in the left thalamus, bilateral supplementary motor area (SMA), and cerebellum in patients with DCM (Figure 2, Table 3). In comparison, increased GMV was observed in the left inferior occipital cortices.

**Table 3:** Correlation coefficients among gFCD, GMV, and clinical measures.

**Brain regions**	**lIOG**	**rIOG**	**Cv**	**rSMA**	**lSMA**	**lTHA**	**lSFG**
**Correlation between GMV and gFCD**							
*R*-value	0.2	0.24	0.31*	0.15	0.17	0.37*	0.34
**Correlation between GMV and JOA**							
*R*-value	0.15	0.17	−0.07	0.06	0.1	0.15	−0.14
**Correlation between GMV and disease duration**							
*R*-value	−0.09	0.14	−0.09	0.14	0.17	0.21	0.13
**Correlation between gFCD and JOA**							
*R*-value	0.19	0.31*	0.20	0.33*	0.31*	0.17	0.15
**Correlation between gFCD and disease duration**							
*R*-value	−0.17	0.15	0.09	0.07	−0.01	0.08	0.17

*lIOG, left inferior occipital gyrus; rIOG, right inferior occipital gyrus; Cv, cerebellum vermis; rSMA, right supplementary motor area; lSMA, left supplementary motor area; lTHA, left thalamus; lSFG, left superior frontal gyrus; GMV, gray matter volume; gFCD, global functional connectivity density*.

*Uncorrected P-value < 0.05. No P-values can survive multiple comparison correction.

### Associations Between Spatial Distributions of gFCD and GMV

As previously mentioned, gFCD increases were primarily located in visual cortices and cerebellum. The gFCD decreases were primarily located in the sensorimotor regions, e.g., bilateral SMA, left thalamus, and left superior frontal gyrus. GMV decreases were observed in the left thalamus, bilateral SMA, and cerebellum in patients with DCM, while GMV increases were observed in the left inferior occipital cortices. Notably, the aberrant patterns of gFCD and GMV differ between regions. In the cerebellum, gFCD increased while GMV decreased, whereas in bilateral SMA and left thalamus, the decreased gFCD was accompanied by decreased GMV. In the left inferior occipital cortices, increased gFCD was accompanied by increased GMV. These findings implied a complex association between gFCD aberrations and GMV changes in DCM. We also performed correlation analysis to further investigate whether gFCD correlates with GMV changes. Multiple regions showed no statistical correlation between gFCD and GMV despite simultaneously being affected, either in patients with DCM or healthy controls.

### Association Between gFCD/GMV and Clinical Variables

No statistical correlation was found between gFCD or GMV and illness duration or preoperative JOA scores. The detailed information can be found in Table 3. The reason was that despite several variables being significantly correlated, i.e., *P* < 0.05, these *P*-values could not survive the multiple comparison correction.

## Discussion

In this study, we first applied the voxel-wise graph theory to investigate the spatial distribution patterns of gFCD and GMV alterations in patients with DCM. According to the results, gFCD and GMV were both affected in some regions of the visual system, sensorimotor cortices, and cerebellum, which are the key regions contributing to the pathology of DCM as indicated by several lines of evidence (15–17, 19, 24). More importantly, our findings showed that decreased gFCD was found in areas primarily located in the sensorimotor cortices, while increased gFCD was observed primarily in areas located in the visual system and cerebellum. Decreased GMV was observed in the left thalamus, bilateral SMA, and cerebellum in patients with DCM, while increased GMV was observed in the left inferior occipital cortices. Furthermore, gFCD and GMV were both affected in most of these regions, but the spatial distribution pattern of gFCD and GMV differed between regions. gFCD and GMV consistently decreased in regions other than the bilateral SMA and left thalamus and consistently increased in regions other than the left inferior occipital cortices. In the sensorimotor cortex, gFCD and GMV alterations were increased in DCM patients, with increased gFCD and increased GMV simultaneously observed in these regions.

### Altered Brain Function and Structure Within the Sensorimotor Cortex Indicated Cortical Compensatory Changes Following Deficits of the Corticospinal Tract in DCM Patients

Our current findings are consistent with previous findings that patients with DCM exhibited gray matter atrophy within the sensorimotor cortex (47, 48, 53). Studies have shown that DCM patients exhibited decreased gray matter density within the primary motor cortex (M1), primary sensory cortex (S1), and supplementary motor area (SMA) (54). Moreover, several studies have also reported decreased regional functional activity and metabolic demand within these regions, indicating that the altered brain function in DCM patients could result from structural changes in the sensorimotor cortices (16, 20, 23). These findings showed that the neural deficits following chronic spinal cord injury further disrupt the signal transmission along the corticospinal tract, and the brain gradually adapted to these alterations and formed a series of compensatory changes at the cortical level.

### The Cerebral Compensatory Changes Were Complex and Appeared in a Wide Range of Cerebral Regions Not Limited to the Sensorimotor Network

Compared with healthy controls, patients with DCM also exhibited altered regional function and functional connectivity within the visual network. The visual cortices, including the primary visual cortex (calcarine gyrus), and the secondary visual cortex (occipital lobe), exhibited significantly increased functional connectivity in patients with DCM (16, 17). These functional alterations were significantly correlated with visual acuity in patients with DCM (16). A resting-state fMRI study was conducted using the dynamic causal model to explore changes in the primary visual cortex, secondary visual cortex, and cerebellum. Their findings showed that patients with DCM exhibited higher bidirectional effective connectivity between the secondary visual cortex and cerebellum, and the increased effective connectivity was correlated with the prognosis of DCM patients (15). Therefore, the adaptive changes following myelopathy play a key role in the neuropathology of DCM. The gFCD increases within the occipital lobe and cerebellum of DCM patients further support previous findings (15–17, 19). In conclusion, the brain compensatory changes in DCM patients were complex and appeared in a wide range of brain regions of multiple brain networks.

### The Brain Structural Alterations Exhibited Indirect Relationships With Functional Abnormalities in DCM Patients

According to the results of this study, the association between anatomical changes and functional activity aberrations is complex and has not been fully elucidated. Previous studies reported that structural deficits do not directly relate to functional abnormalities (55, 56). Longitudinal studies aiming to evaluate the onset of connectivity abnormalities as DCM develops should be conducted to explore the nature of associations between the structural deficits and functional connectivity alterations in DCM (56). Taken together, these results implied that aberrant gFCD and GMV are independent characteristics of DCM. Furthermore, no association between aberrant gFCD/GMV and clinical measures was detected in our current study, which is consistent with findings from previous studies (57). Thus, illness duration and symptom severity are not linearly correlated with brain functional or structural alterations (57, 58). In terms of clinical significance, imaging biomarkers are easily acquired for non-invasive measures in clinical practice, which could prove suitable for monitoring the development of DCM. However, this does not necessitate routine brain rs-fMRI for all DCM patients, especially those with progressive myelopathy, i.e., those needing immediate decompression surgery. However, patients with a long history of myelopathy or mild spinal cord compression but no clinical symptoms make it difficult for surgeons to decide whether surgical treatment is justified. Such brain rs-fMRI analyses may have potential utility for monitoring the progression of myelopathy, thus aiding clinical decision making.

### Limitations

Our current study has several limitations. Firstly, no postoperative fMRI data were collected due to the possible artifacts and heating problems caused by surgical implants. We will collect these data when it is safe in the future. Secondly, all the patients included underwent long-term conservative intervention before enrolling in our study. Therefore, studies including drug-naïve patients with DCM are also warranted in the future. Thirdly, our current study only analyzed gFCD, while other resting-state fMRI metrics such as the amplitude of low-frequency fluctuations (ALFF) and regional homogeneity (ReHo) are also worth analyzing. Fourthly, our current study only included 35 patients, a relatively small sample. Future studies enrolling a larger sample are needed. Finally, our univariate approach could detect the differences in the amplitude of brain variables between patients and healthy controls but could not fully describe the spatial pattern differences between patients and controls. Therefore, multi-variate approaches are needed for interpreting these associations.

## Conclusion

The present study provided new evidence on aberrant gFCD and decreased GMV in multiple brain regions associated with the DCM pathology. The results indicate that gFCD and GMV are simultaneously affected in multiple regions. However, their spatial distribution patterns in the intergroup difference maps were different. These findings suggest that structural and functional alterations may independently contribute to the neurobiology of DCM. However, the nature of the associations between the structural deficits and functional alterations should be explored through longitudinal studies to determine the onset of connectivity abnormalities as DCM develops.

## Data Availability Statement

The raw data supporting the conclusions of this article will be made available by the authors, without undue reservation.

## Ethics Statement

The studies involving human participants were reviewed and approved by Xiangyang Central Hospital. The patients/participants provided their written informed consent to participate in this study.

## Author Contributions

YZ collected the data and wrote the manuscript. JS revised the manuscript and analyzed the data. Both authors contributed to the article and approved the submitted version.

## Conflict of Interest

The authors declare that the research was conducted in the absence of any commercial or financial relationships that could be construed as a potential conflict of interest.

## Publisher's Note

All claims expressed in this article are solely those of the authors and do not necessarily represent those of their affiliated organizations, or those of the publisher, the editors and the reviewers. Any product that may be evaluated in this article, or claim that may be made by its manufacturer, is not guaranteed or endorsed by the publisher.
